# Comparative antibacterial effects of ginger and marjoram extract versus conventional irrigants on mature *Enterococcus faecalis* biofilms: An *in vitro* study

**DOI:** 10.4317/jced.60081

**Published:** 2023-04-01

**Authors:** Hadi Mokhtari, Mahsa Eskandarinezhad, Mohammadhossein-Soruosh Barhaghi, Solmaz Asnaashari, Fatemeh-Yeghaneh Sefidan, Atefeh Abedi, Sepideh Alizadeh

**Affiliations:** 1 Department of Endodontics, Faculty of Dentistry, Tabriz University of Medical Sciences, Tabriz, Iran; 2 Immunology Research Centre, Tabriz University of Medical Sciences, Tabriz, Iran; Drug Applied Research Centre, Tabriz University of Medical Sciences, Tabriz, Iran; Department of Microbiology, Faculty of Medicine, Tabriz University of Medical Sciences, Tabriz; 3 Biotechnology Research Center, Tabriz University of Medical Sciences, Tabriz, Iran; 4 Department of Bacteriology and Virology, School of Medicine, Tabriz University of Medical Sciences, Tabriz, Iran; 5 Faculty of Dentistry, Tabriz University of Medical Sciences, Tabriz, Iran

## Abstract

**Background:**

This study evaluated antibacterial effects of Ginger and Marjoram extract compared with Routine Intracanal Irrigants on Mature *Enterococcus faecalis* Biofilms.

**Material and Methods:**

Sixty-six extracted human teeth, were randomly assigned to four groups 5.25% sodium hypochlorite (NaOCl), 2% chlorhexidine (CHX), chloroform extract of marjoram (Origanum majorana), and oil extract of ginger (Zingiber officinale), and two positive and negative control groups (n=11). Samples were contaminated with *E. faecalis*, except the negative control group. Then the root canals were irrigated with solutions above, after which dental debris was collected from each tooth separately, followed by culturing on plates containing BHI agar. The bacterial counts were finally determined with a colony counting machine.

**Results:**

No bacterial growth was detected in the NaOCl, CHX, and negative control groups. However, some bacterial growth was observed in the ginger and marjoram groups. All four solutions successfully eliminated *E. faecalis* biofilms compared to the positive control group. Significant difference in the median bacterial growth between the ginger and marjoram groups and the positive control group (*P*<0.001) has been shown. There was no significant difference in median bacterial growth between the ginger and marjoram groups (*P*=0.94).

**Conclusions:**

Chloroform extract of marjoram and oil extract of ginger were effective in eliminating 6-week-old biofilms of *E. faecalis* *in vitro*; however, their effect was not as favorable as CHX and NaOCl. These herbal extracts yielded promising results in the present study; therefore, they can be used as intracanal irrigants instead of chemical agents.

** Key words:**Biofilm, Chlorhexidine, E. faecalis, Ginger, Marjoram, Sodium hypochlorite.

## Introduction

Several studies have reported the prevalence of *E. faecalis*, a gram-positive facultative anaerobic bacterial species, in endodontically treated teeth with persistent or secondary infections that require retreatment (1). One of the most essential characteristics of this bacterial species is its ability to form biofilms.

A biofilm is an organized structure of bacteria that is more resistant to chemical agents than the planktonic state (2). Although mechanical and chemical techniques are used to eliminate microorganisms from the root canals, some bacteria might persist in the root canal. Therefore, bioactive substances, different irrigation solutions and intracanal medicaments are used between treatment sessions (2,3). Sodium hypochlorite (NaOCl) solution has been used as an irrigation solution in endodontic treatment since 1920. The antimicrobial properties of this solution are attributed to hypochlorous acid. Some disadvantages of NaOCl include its toxicity in contact with periradicular tissues, inability to remove the smear layer, and induction of corrosion in metallic products. The odor and flavor of NaOcl are unfavorable for patients, and its vapor irritates the eyes(4,5). CHX has been introduced as an intracanal irrigation solution due to its broad antimicrobial activity and substantivity (6,7). CHX is bacteriostatic at low concentrations and bactericidal at high concentrations (8). This chemical agent has no tissue-dissolving properties contrary to NaOCl (9,10).

Considering the side effects and limitations of chemical agents, in recent years, the biological properties and antibacterial effects of herbal medicines have been an active research field. The antibacterial properties of herbal medicines are mainly attributed to the phenolic components in the plant extracts, including thymol, carvacrol, and eugenol (11).

Marjoram is a multipurpose aromatic plant species that primarily grows in Mediterranean regions (12). Coccimiglio reported the antibacterial activity of the ethanolic extract of marjoram on gram-negative and gram-positive bacterial species, which was attributed to the phenolic isomers of thymol and carvacrol (13).

Ginger is a medicinal plant widely used in spices and drinks. On the other hand, this plant is used in the treatment of many diseases. Some ginger derivatives have antioxidative, anti-inflammatory, antiviral, and anti-platelet properties that activate the immune system (14,15). The active chemical ingredients of this plant are phenolic components, including gingerols and shogaols (15). Since no study has evaluated and compared the antibacterial effects of 5% oil extract of ginger and 1% chloroform extract of marjoram with routinely used root canal irrigation solutions on mature *E. faecalis* biofilms, the present study was undertaken to compare the materials mentioned above.

## Material and Methods

This study was approved by the Ethical Committee of Tabriz University of Medical Sciences, Tabriz, Iran (IR.TBZMED.REC.1399.136).

Sixty-six patients referred for tooth extraction for orthodontic treatment or periodontal problems were selected and evaluated.

The inclusion criteria consisted of the following: one-root teeth, a single root canal, no root surface caries, no calcification, no internal or external root resorption, no previous root canal treatment, no cracks or traumas, no anomalies, and a lack of severe curvature.

To determine the sample size, the results of a study by Kakare *et al*. were considered, and the mean no growth inhibition zones of 12.66±2.05 and 10.66±8.94 in the NaOCl and plant extract groups were considered, respectively. The type I error was set at α=0.05, with a study power of β=80, and the sample size was increased by 20% (16). Finally, 11 samples were included in each group, adding up to 66 pieces.

All the teeth were cleaned with a chisel and curette to remove soft and hard tissue remnants and surface debris. The samples were then immersed in 0.5% NaOCl (Morvabon, Tehran, Iran) for 24 hours for surface disinfection, followed by storage in normal saline solution (Injection and Pharmaceutical Products Company, Tehran, Iran) at room temperature until the tests were carried out.

The samples were decoronated at the cementoenamel junction (CEJ) using a long cylindrical carbide bur in a high-speed handpiece under copious irrigation, perpendicular to the tooth long axis to achieve a standard root length of 12 mm. Then a barbed broach proportionate to the root canal diameter was used to remove the pulp remnants. Then a #15 K-file (Mani, INC, Tochigi, Japan) was inserted into the root canals so that its tip was visible at the apical foramen. Then the working length (WL) was determined to be 1 mm short of this length.

All the root canals were prepared manually. Root canal filling was continued up to file #40, and flaring was carried out manually up to file #60 using the step-back technique; 2 mL of normal saline solution (Injection and Pharmaceutical Products Company, Tehran, Iran) was used for root canal irrigation with a 30-G syringe needle (Avapezeshk, Tehran, Iran), and 5 mL of normal saline solution was used as a final rinse. After the final irrigation of each root canal, each sample was irrigated with 5 mL of 5.25% NaOCl solution (Morvabon, Tehran, Iran), followed by 5 mL of 17% EDTA (Diadent, Seol, Korea) to remove all the debris and the smear layer. The root canals were sealed with cyanoacrylate glue (MxBon, Yi Hsien, Chia, Taiwan) to prevent bacterial microleakage. The tooth surfaces were sealed with nail varnish.

**Figure 1:**
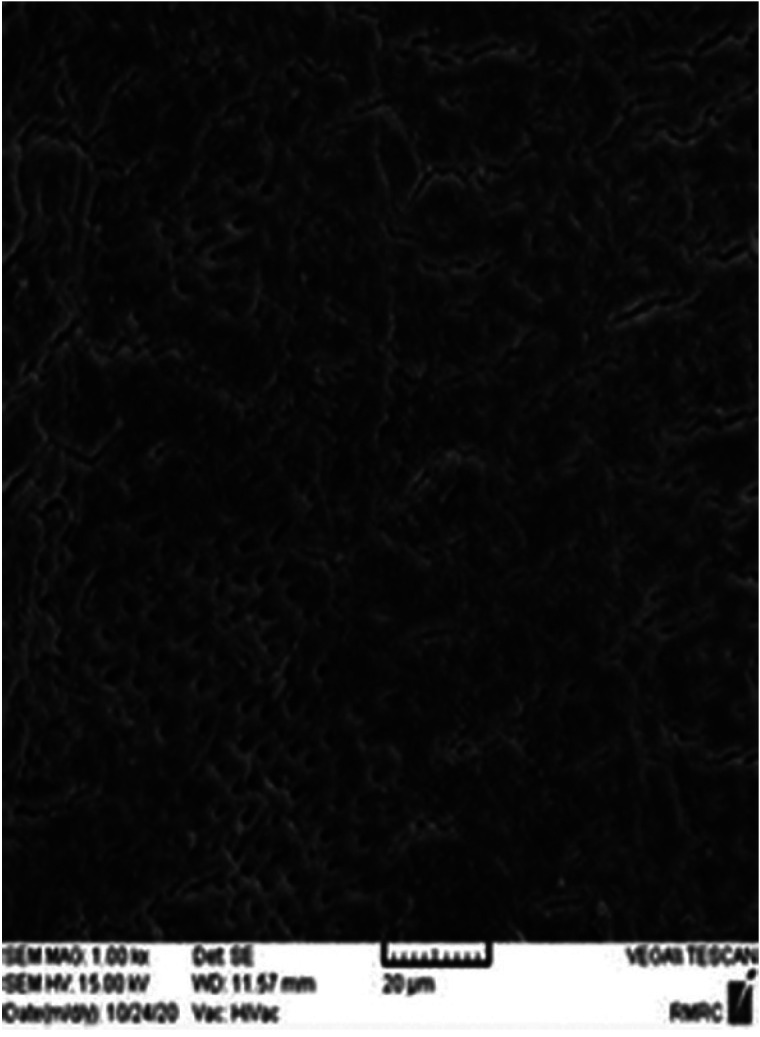
SEM images. ×100 magnification; absence of *E. faecalis* biofilms and the smear layer.

**Figure 2:**
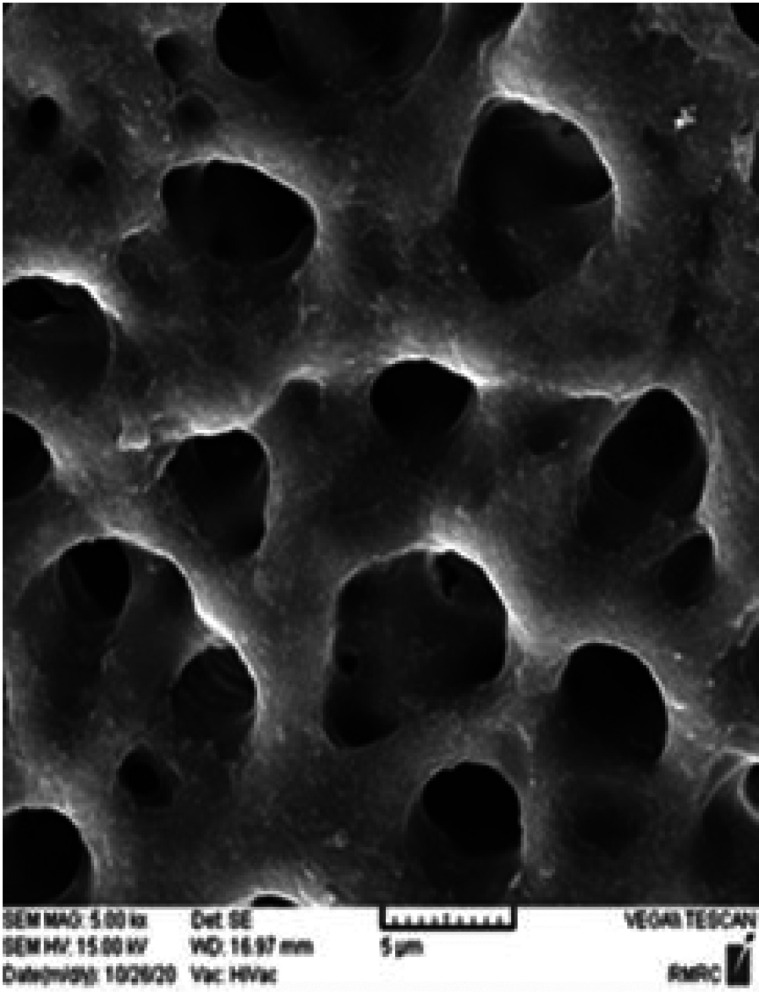
×500 magnification; absence of *E. faecalis* biofilms and the smear layer.

**Figure 3:**
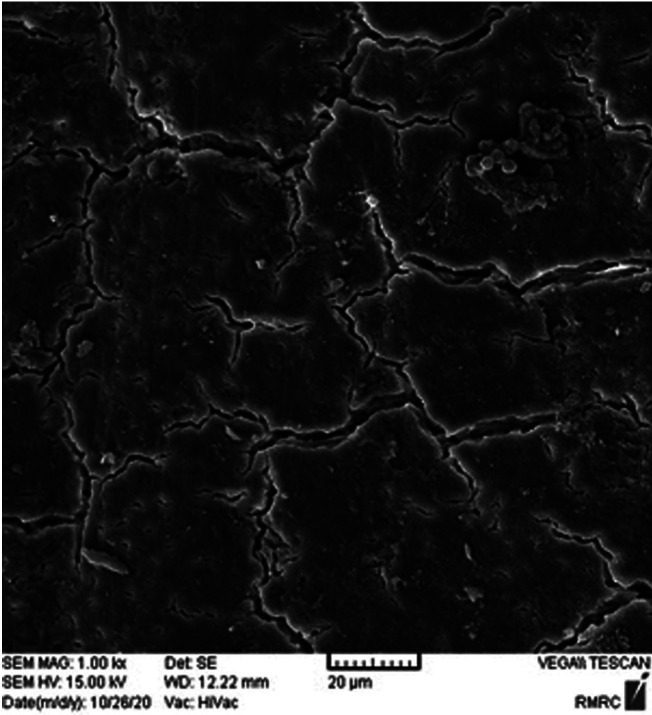
SEM images. ×100 magnification; the presence of mature *E. faecalis* biofilms.

**Figure 4:**
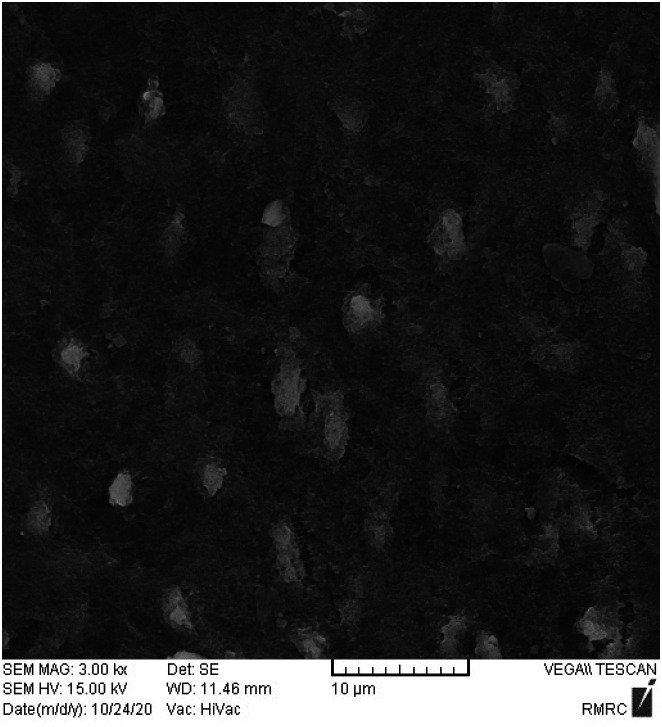
×500 magnification; the presence of mature *E. faecalis* biofilms.

The samples were autoclaved for sterilization at 121ºC for 15 minutes. Then all the samples were contaminated with *E. faecalis* except for the negative control group. To this end, the standard strain of *E. faecalis* (ATCC29212) was cultured in BMI agar plates under sterile conditions and incubated at 37ºC for 24 hours. Then 0.5 standard McFarland concentration (1-2×108 CFU/mL) in the BH/I culture was prepared from that strain. Under sterile condition, the prepared concentration was transferred to each microtube containing the tooth followed by incubation at 37ºC. The microbial contents were evaluated every day and replaced with fresh standard suspension. The process was repeated for six weeks. The bacterial suspension was added to all the microtubes except for the negative control group. Eleven microtubes containing the negative control teeth remained intact without bacterial injection. After six weeks, when mature biofilms formed in all the microtubes except for the negative control group, in the next stage, to confirm intracanal contamination and bacterial concentration, 11 samples consisted of positive control without using irrigation solutions. To ensure biofilm formation, two samples from each positive and negative control group were randomly selected and evaluated under a scanning electron microscope (SEM). Evaluation was performed in Razi Metallurgical Research Center in Tehran, (Figs. 1-4).

Subsequently, the samples were randomly assigned to six groups (n=11), in which mature biofilm had formed (except for the negative control group) were irrigated with one of the irrigation solutions, as follows.

Group 1: Teeth #1 to #11 were irrigated with the oil extract of ginger.

Group 2: Teeth #12 to #22 were irrigated with the chloroform extract of marjoram.

Group 3: Teeth #23 to #33 were irrigated with 5.25% NaOCl solution (Morvabon, Tehran, Iran).

Group 4: Teeth #34 to #44 were irrigated with 2% CHX (Morvabone, Tehran, Iran).

Group 5: Teeth #45 to #55 (the negative control group): without bacterial suspension injection, irrigation with root canal irrigation solution.

Group 6: Teeth #56 to #66 (the positive control group): injection of bacteria, no irrigation solution.

Preparation of chloroform extract of marjoram and oil extract of ginger

The marjoram extract was prepared using the maceration or wetting method by adding around 100 gr of the dry powder of the plant (Drug Applied Research Center, Tabriz, Iran) in a container, and the appropriate solvent, i.e., chloroform, was added to it and mixed well. Then, the solution was kept away from light at a proper temperature for 4 hours, and the supernatant was removed, followed by adding the solvent again. This process was repeated 3-4 times to separate the materials dissolved in the solvent. Finally, the collected solution was dried in a rotary evaporator, and the dry extract was used for the tests. This way, 1% chloroform extract of marjoram was prepared (17).

The ginger was prepared with a Clevenger machine using the water distillation method. Around 5000 gr of ginger rhizome powder (Drug Applied Research Center, Tabriz, Iran) was transferred into the Erlenmeyer flask in the Clevenger machine and mixed with a proper volume of water. Then, the solution underwent heat in the water distillation method, and the oil extract was collected (18).

All the root canals in all the study groups were irrigated with 1 mL of the relevant solution using a 3-mL syringe with a 30-G needle for 2 minutes. The needle was inserted into the root canal until it did not penetrate further and then pulled back a little to avoid being locked in the root canal, followed by injection.

Finally, all the root canals were irrigated with 5 mL of sterile distilled water to remove the remnants of the medications and the irrigation solutions. Under sterile conditions, #5 and #4 Gates-Glidden drills (Mani, INC, Tochigi, Japan) were interested into the root canals once using low pressure, and debris and dentin chips were collected from the dentin root canal length. Then the debris was transferred separately into sterile microtubes. The contents of each microtube were diluted in sterile physiologic serum three times (100 μL of the contents and 900 μL of normal saline solution in new microtubes), and 100 μL from each dilution was selected in BHI agar plates for bacterial growth and colony formation, followed by incubation at 37ºC for 24 hours. The colony counts were determined after incubation.

To count the number of bacterial colonies in each tube (samples) several dilutions (10 -1, 10 -2, 10 -3, 10 -4 …) were prepared. Ten microliter of dilute microbial suspension was inoculated on the surface of BHI agar plate and then spread with a sterile bent-glass rod by the spread plate

technique and incubated for 24 hours at 37°C. After the incubation, the plate that containing 30–300 cells were selected, the number of colonies was counted and then was reported in CFU/mL.

The data were reported using median descriptive statistics (first quartile, third quartile). The normality of data was analyzed with the Kolmogorov–Smirnov test. Since the data were not distributed normally, the Kruskal-Wallis test was used to compare CFU counts between the different study groups. Mann-Whitney test was used for two-by-two comparisons of the groups. SPSS 17 was used for statistical analyses of the data at a significance level of *P*<0.05.

## Results

The colony count results were as follows. In groups 1 and 2, i.e., the oil extract of ginger and chloroform extract of marjoram, of 11 plates evaluated, three plates did not exhibit bacterial growth, and the median of the number of colonies formed in the remaining plates in group 1 was 110, with 9.50 units in the remaining plates in group 2; therefore, the bacterial colony formation percentage in these solutes was <1%.

In none of the samples in group 3 (5.25% NaOCl) and group 4 (2% CHX), and group 5 (negative control), colony formation observed on the BHI agar plates. However, in 10 plates in group 6 (positive control), a large number of colonies were observed.

The results of the Kruskal-Wallis test showed that there was a statistically significant difference between the median colony formation in the ginger, marjoram and positive control groups (*p-value*<0.001), (Table 1.)

**Table 1:**
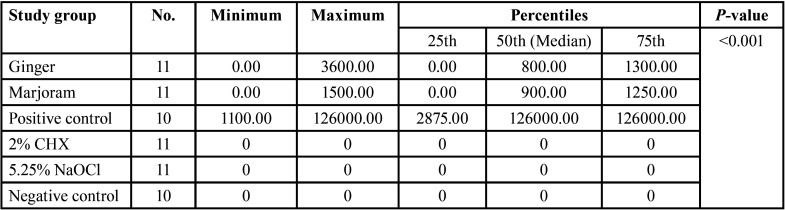
Antimicrobial activity of ginger and marjoram against Enterococcus faecalis biofilm determined by colony-forming units.

On the other hand, the Mann-Whitney non-parametric test showed that there was no statistically significant difference between the median colony formation number of bacteria in the ginger and marjoram groups (*p-value*=0.94), but the difference between the median colony formation number of bacteria between ginger and the positive control was significant (*p-value*<0.001) and also the difference between the median colony formation number between marjoram and positive control was significant (*p-value*<0.001), (Table 2.)

**Table 2:**
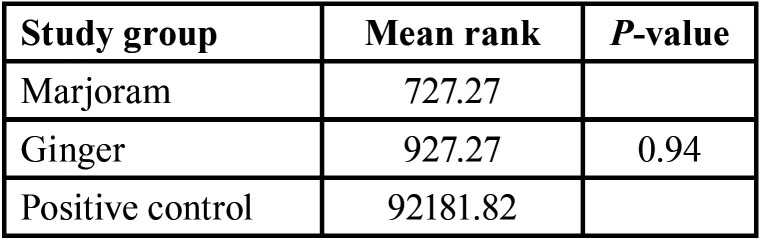
Mean colony-forming unit in the ginger and marjoram groups.

## Discussion

The key to successful root canal treatment is the mechanical and chemical debridement of the infected root canal system (19).

The most commonly isolated bacterial species from the root canal treated teeth with periradicular lesions is *E. faecalis* (20).

In the present study, 5.25% NaOCl, 2% CHX, chloroform extract of marjoram, and oil extract of ginger were used as root canal irrigation solutions. According results of this study, 5.25% NaOCl and 2% CHX showed the highest antibacterial efficacy when compared to that of marjoram, and ginger extract. On the other hand, the bacterial colony formation percentage in herbal groups solutes was <1%. An ideal root canal irrigation solution should have antibacterial activity and tissue-dissolving properties with minimal toxicity (21).

Herbal medicinal products have attracted attention in traditional medicine since antiquity. Herbal medicines are used in dentistry as analgesics, anti-inflammatory agents, antibiotics, and root canal irrigation solutions in root canal treatment procedures (22).

The principal components of ginger are volatile oils, gingerols, shogaols, and diarylheptanoids, with antibacterial, antioxidative, and anti-inflammatory properties (23). Marjoram, too, is widely used in traditional medicine. Fifty-five ingredients have been identified in its volatile oil extract, with carvacrol as the main ingredient (86.5%) (24).

Many studies have shown that carvacrol and thymol are potent antimicrobial agents against gram-positive and gram-negative bacteria (25). Volatile oils rich in phenolic components in this plant have high antibiotic activity (24).

Ahmed *et al*. evaluated ginger’s antimicrobial efficacy and emphasized that ginger, against Gram-positive bacteria including *Enterococcus Faecalis*, is an effective antimicrobial herb (26).

Benbelaid el al. evaluated some essential oils including Origanum in treatment of infections caused by multidrug-resistant *E. faecalis*. They reported that capitata and Origanum were the most active ones and can be used in endodontic treatments especially persistent endodontic infections (27).

Paudel *et al*. reported that majorana from two different locations in Nepal, possessed moderate antibacterial activity against some gram-positive bacteria, however it demonstrated considerable activity against the fungal strains (28).

Shantini *et al*. compared the antibacterial effect of cinnamon and garlic as effective irrigation solutions on *E. faecalis* *in vitro* and reported that 2% CHX and 5.25% NaOCl had thorough inhibitory effects on the samples; however, although cinnamon and garlic were favorably effective, they were not as ideal as the chemical solutions, consistent with the present study (29).

Evren *et al*. evaluated the antibacterial properties and smear layer-removing effect of oregano and reported no significant differences between 5% CHX and 2% marjoram extract in eliminating *E. faecalis*. In addition, the 1% extract of marjoram and 1% NaOCl exhibited similar antibacterial effects; these materials were better than the normal saline solution and EDTA but were not as effective as CHX (30). Based on the results of the study above, CHX was the most effective solution. In addition, different concentrations of marjoram extract alone could not successfully remove the smear layer from the dentin surfaces; however, this extract was effective in removing the smear layer without wearing the dentin when combined with EDTA, which is different from the present study. Such a discrepancy in the results might be attributed to differences in therapeutic concentrations or the different geographical origins of marjoram.

Makawa *et al*. evaluated the antimicrobial effect of ginger extract and the oil extract of propolis on bacteria and their endotoxins in root canals. 2% CHX gel exhibited the highest efficacy in neutralizing the endotoxins of microorganisms and the minimum effect on *E. faecalis*, which is different from the present study (31). This discrepancy in the results might be attributed to the various drug forms of CHX gel used as an intracanal medicament and not as an intracanal solution.

## Conclusions

Based on the results, 2% CHX, 5.25% NaOCl, 1% marjoram, and 5% ginger extract exhibited significant inhibitory effects on *E. faecalis* biofilm formation, which was 100% in the chemical agent groups and acceptable in the plant extract groups. There was no significant difference in the inhibitory effect on *E. faecalis* biofilm formation between the ginger and marjoram groups. Therefore, ginger and chloroform extract of marjoram can be considered effective herbal solutions for root canal irrigation.

However, considering the significant differences between the *in vitro* and clinical conditions, further clinical studies are necessary based on the *in vitro* conditions of the present study, especially in terms of concentrations and different plant forms and also using more complex biofilms, to evaluate the biocompatibility of ginger and marjoram with periradicular tissues and evaluate them in different depths of dentinal tubules to reach more accurate conclusions.
